# Effect of individualized PEEP on lung ultrasound score and optic nerve sheath diameter in elderly patients undergoing laparoscopic rectal cancer surgery: A randomized controlled trial

**DOI:** 10.1371/journal.pone.0328067

**Published:** 2025-08-08

**Authors:** Furong Bai, Hong Yin, Shuang Zhang, Daneng Wei, Jiansheng Wang, Mingliang Yi

**Affiliations:** 1 Department of Anesthesiology, Chengdu Fifth People’s Hospital (Affiliated Fifth People’s Hospital of Chengdu University of Traditional Chinese Medicine), Chengdu, China,; 2 Center for Medicine Research and Translation, Chengdu Fifth People’s Hospital (Affiliated Fifth People’s Hospital of Chengdu University of Traditional Chinese Medicine), Chengdu, China; University of Birmingham, UNITED KINGDOM OF GREAT BRITAIN AND NORTHERN IRELAND

## Abstract

**Objective:**

Positive end-expiratory pressure (PEEP) is widely used during surgery, but its effects on lung and brain protection remain debated. This study aimed to evaluate the impact of individualized PEEP on lung ultrasound score (LUS) and optic nerve sheath diameter (ONSD) in elderly patients undergoing laparoscopic rectal cancer surgery.

**Methods:**

Forty-six patients aged 60–79 years undergoing laparoscopic rectal tumour resection between June 2022 and December 2022 were randomized into two groups: Group E (individualized PEEP guided by driving pressure) and Group C (control group, PEEP = 5 cm H_2_O). LUS was assessed 30 minutes postoperatively. ONSD was measured at 5 minutes before anesthesia induction (T0), 5 minutes after tracheal tube insertion (T1), 5 and 60 minutes after Trendelenburg positioning (T2, T3), and 30 minutes postoperatively (T4). Arterial oxygen index (OI) and arterial partial pressure of carbon dioxide (PaCO_2_) were recorded post-intubation and pre-extubation. Postoperative pulmonary and neurological complications were followed up.

**Results:**

Postoperative LUS was significantly lower in Group E than in Group C (*P* < 0.05). OI was significantly higher in Group E before extubation (*P* < 0.05). There were no significant differences in ONSD between groups. Within each group, ONSD values at T2 and T3 were significantly higher than those at T0 (*P* < 0.01). No significant differences were observed in the incidence of postoperative complications between the two groups.

**Conclusions:**

During laparoscopic radical resection for rectal cancer, individualized PEEP reduces LUS scores, improves oxygenation, and does not increase ONSD values compared to fixed PEEP.

**Trial registration:**

Chinese Clinical Trial Registry: ChiCTR2200060434.

## Introduction

Laparoscopic radical resection of rectal cancer is more common in elderly patients. In addition to establishing pneumoperitoneum, the patient must be placed in the Trendelenburg position during the operation. However, advanced age, pneumoperitoneum, the Trendelenburg position and general anaesthesia are all high-risk factors for postoperative pulmonary complications (PPCs) and atelectasis [1–3]. Preventing these complications is crucial for patient recovery and reducing hospital stays [4,5]. Additionally, pneumoperitoneum and Trendelenburg positioning may increase intraocular and intracranial pressure, potentially leading to neurological complications [6,7].

Studies have shown that a lung-protective ventilation strategy (LPVS) can reduce the incidence of PPCs [8,9]. LPVS involves setting the tidal volume and positive end-expiratory pressure (PEEP), as well as the oxygen concentration. During mechanical ventilation,it also involves applying lung recruitment manoeuvres and allowing hypercapnia [8,9]. PEEP is an essential part of LPVS; however, optimal PEEP levels vary among individuals [10]. Although individualised PEEP guided by driving pressure (ΔP) can optimise respiratory mechanics and reduce the incidence of PPCs [11,12], it is unclear whether ΔP-guided PEEP increases intracranial pressure (ICP) and neurological complications. Ultrasound measurement of ONSD is a reliable method for evaluating ICP [13], and lung ultrasound score (LUS) can evaluate lung conditions (the higher the score, the more severe the atelectasis) [14,15]. This study hypothesised that individualised PEEP guided by ΔP could reduce LUS and ONSD and the incidence of PPCs. This would provide a basis for lung- and brain-protective ventilation strategies in laparoscopic surgery.

## Methods

### Participants

This trial was approved by the ethics committee with number AF/54/2020-02.3. All participants had signed the written informed consent. The trial recruitment started on June 6, 2022 and ended on December 11, 2022. 46 patients undergoing laparoscopic radical resection of rectal cancer under general anesthesia (aged 60 ~ 79 years, BMI 18 ~ 24 kg/m^2^, American Society of Anesthesiologists (ASA) I–III) were randomly divided into control and experimental groups based on the random numbers. Exclusion criteria were as follows: Patients with eye and brain diseases (including high ICP and high IOP); History of eye, craniocerebral, and thoracic surgery; Preoperative respiratory diseases such as pulmonary bullae, pneumothorax, and history of pneumonia; Unable to communicate and important organ dysfunction.

### Randomization and blinding

The random numbers were computer-generated and randomly divided into two groups at a ratio of 1:1, placed in opaque sealed envelopes that were handed to the anesthesiologist by a non researcher. The anesthesiologist did not open the envelope until induction of anesthesia. All LUS and ONSD assessments were performed by a single investigator blinded to group allocation, following a standardized protocol.

### Anesthesia and monitoring

HR, non-invasive blood pressure (NIBP),oxygen saturation (SpO_2_), and bispectral index (BIS) value were routinely monitored in all patients after entering the operating room. Under local anesthesia, the radial artery was punctured for pressure measurement and blood gas analysis. Anesthesia induction: cis-atracurium 0.15 mg/kg, sufentanil 0.3 μg/kg, etomidate 0.2 mg/kg intravenous injection. After 5 minutes, the tracheal tube was inserted at the appropriate depth and fixed. Anesthesia was maintained by inhalation of 1 ~ 2% sevoflurane for sedation, intravenous pumping of 0.1 ~ 0.2 μg/kg·min remifentanil for analgesia, intermittent intravenous injection of cisatracurium (3 ~ 5 mg) as needed for muscle relaxation, and the BIS value was maintained at 40 ~ 60. The mechanical ventilation parameters setting:tidal volume was 6 mL/kg, respiratory rate was 12–18 bpm, inspiratory/expiratory ratio was 1:2. PETCO_2_ maintained at 35 ~ 50 mmHg. Trendelenburg position was controlled at 30 degrees and pneumoperitoneum pressure was controlled within 12 mmHg for all patients during the operation. Lung recruitment maneuvers were performed every 30 minutes. Dezocine (5 mg) was intravenously injected 30 minutes before the end of surgery. All patients received patient-controlled intravenous analgesia (PCIA): basal infusion dose, sufentanil (2 µg/h), butorphanol (60 µg/h); bolus dose, sufentanil (0.5 µg), butorphanol 15 µg; 15 minutes lock time.

#### Intervention.

In the control group, PEEP was set at 5 cmH_2_O, whereas in the experimental group, 5 cmH_2_O PEEP was used immediately after tracheal intubation. After pneumoperitoneum was established, PEEP was gradually increased by 1 cmH_2_O every 10 respiratory cycles in the Trendelenburg position until ΔP reaches a minimum value or ΔP does not increase but the peak inspiratory pressure (Ppeak) has increased to 28 cmH_2_O.

### Stopping rules

PEEP was stopped immediately if heart rate or mean arterial pressure fluctuated by more than 20% of the baseline value or an arrhythmia occurred. When systolic blood pressure or mean arterial pressure was lower than 20% of its basic value, ephedrine (3 ~ 6 mg) was injected intravenously. When the HR was lower than 50 bpm, atropine (0.3 ~ 0.5 mg) was given intravenously.

### LUS method

Referring to previous literature [16], transthoracic lung ultrasound scanning was performed with Sono ultrasound convex array probe in the supine position. The lungs were divided into 12 regions based on the nipple horizontal, parasternal, anterior axillary, and posterior axillary lines. Each region was scored from 0 to 3 points, and the scores of the 12 examined regions were accumulated into the LUS (0–36 points).

The LUS criteria were as follows: 0, lung sliding is normal and B-lines less than three; 1, B-lines more than three; 2, coalescent B-lines; and 3, consolidated lungs [16]. The LUS was obtained by the same physician who was blinded to the group assignments.

### ONSD measurement method

A linear array probe was used to measure the ONSD. The Sono convex array ultrasound probe was placed in the middle of the eyelid, with no pressure applied to the eye. The ONSD was measured 3 mm posterior to the eyeball in the orbital coronal plane, twice on each side, and the average value was recorded. All measurements were performed by the same investigator, who also blinded to the group assignments.

### Outcomes

The primary outcome was the LUS at 30 minutes after surgery. The secondary outcomes were ONSD at 5 minutes before anesthesia induction (T0), 5 minutes after tracheal tube insertion (T1), 5 and 60 minutes after Trendelenburg positioning (T2, T3), and 30 minutes after surgery (T4); The OI and PaCO_2_ at T0 and before extubation at the end of the operation; and Ppeak, PETCO_2_, HR, and MAP at T1-T3.

### Follow-up

The research staff collected all data until postoperative day 7 or hospital discharge (whichever occurred first),Patients discharged to home before day 7 without complications were considered free of complications at day 7.

### Statistical analysis

The sample size for this trial was estimated based on the primary outcome (LUS). The hypothesis was that using individualized PEEP can reduce the LUS 30 minutes after surgery, and the sample size was calculated based on our pre-test. We assumed that the average LUS value in group C at the end of surgery would be 9.54 ± 1.02, and the experimental group would reduce the LUS to 8.54 ± 1.02. To achieve a power of 0.8, with a two-sided α of 0.05, 18 patients were required in each group with a dropout rate of 20%,needing 23 patients in each group.

The SPSS 26.0 software was used to analyse outcome data. Continuous variables were expressed as median (interquartile range) or mean ± SD. Rate and constituent ratio presented as counts (%). Data with normal distribution was evaluated using the independent sample t-test, and non-normal was calculated using the Mann–Whitney U-test. Rate and constituent ratio were analyzed by chi-square test. The significance level was set at 0.05.

## Results

**Fig 1:**
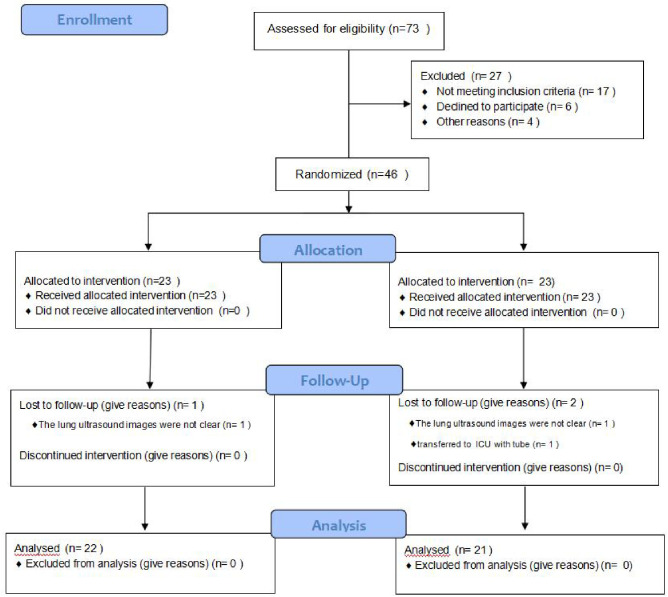
Flow diagram of participant enrollment.

Enrolment ceased once the target sample size had been reached. Between June 2022 and December 2022, a total of 73 patients were assessed. Of these,17 patients did not meet the inclusion criteria, 6 patients refused to participate in the trial, and 4 patients met the exclusion criteria. 46 patients were randomly allocated to group E and group C. However, 2 patients were excluded due to poor ultrasound images, and 1 patient was transferred to ICU due to a long operation time. Finally, 43 patients (22 and 21 in the experimental group and control group, respectively) were analyzed (Fig 1). No significant differences in sex, age, ASA classification, BMI, duration of surgery, fluid infusion, or blood loss (Table 1).

**Table 1:** Baseline characteristics.

Indicator	Group E (n = 23)	Group C (n = 23)	*t/*χ^2^value	*p* value
Age (year)	68.83 ± 5.12	70.65 ± 5.68	−1.144	0.259
Male /Female	18/5	16/7	0.451	0.738
BMI (kg/m^2^)	21.59 ± 1.50	21.37 ± 1.48	0.524	0.603
ASA,II/III	18/5	17/6	0.119	0.730
blood loss (ml)	117.39 ± 23.59	118.26 ± 32.28	−0.104	0.917
Fluid infusion (ml)	2195.65 ± 357.36	2169.57 ± 438.42	0.221	0.826
Duration of Surgery (minute)	258.04 ± 40.86	253.91 ± 53.74	0.293	0.771

*Abbreviations: BMI body mass index, ASA American Society of Anesthesiologists. Chi-square test was used for ASA and gender. The rest were tested by independent sample t-test. (*P* > 0.05).

### Primary outcomes

The LUS scores are presented in Table 2. At 30 minutes after extubation, the LUS in group E was significantly lower than group C, (7 [6–8] vs. 8 [7–8], *P* < 0.05).

**Table 2:** Comparison of LUS, PaCO_2_, and OI between the two groups.

Indicator	Group E (n = 22)	Group C (n = 21)	*p* value
OI	405.68 ± 43.37	373.90 ± 52.41	0.036
PaCO_2_,mmHg	44.71 ± 4.99	46.09 ± 5.31	0.385
LUS [M(IQR)]	7 (6 ~ 8)	8 (7 ~ 8)	0.040

*Independent sample t-test was used for PaCO_2_ and OI.Rank sum test was used for LUS,*P* < 0.05

### Secondary outcomes

The OI and PaCO2 are presented in Table 2. The OI in group E was significantly higher than group C before extubation, (405.68 ± 43.37 VS 373.90 ± 52.41, *P* < 0.05). However, there was no significant difference in the PaCO_2_ levels between the two groups, (44.71 ± 4.99 VS 46.09 ± 5.31, **p* *= 0.385).

**Fig 2:**
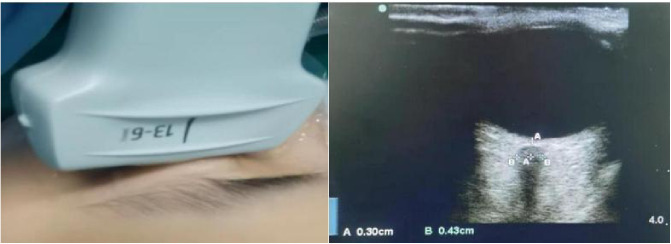
ONSD measurement method.

**Fig 3:**
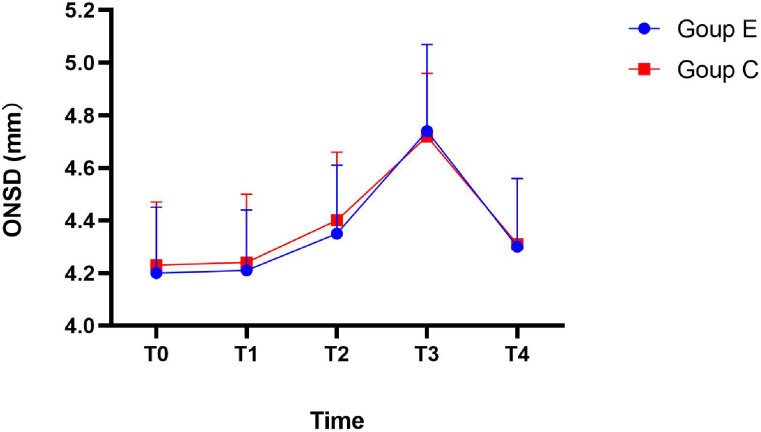
Trend of ONSD over time.

The ONSD (measurement method was shown in Fig 2) are presented in Table 3 and Fig 3. Compared with T0, the ONSDof the two groups increased significantly at T2, T3 (*P* < 0.01). Compared with T1, the ONSD increased significantly at T3 (*P* < 0.01). Compared with T2, the ONSD increased significantly at T3 (*P* < 0.01). However, there were no significant difference in ONSD between the two groups at different time points.

**Table 3:** Comparison of ONSD between the two groups.

Group	Group E (n = 22)	Group C (n = 21)	t	*P*	F_group_	F_time_	F_Interaction_
T0	0.420 ± 0.025	0.423 ± 0.024	−0.381	0.705	0.082	301.865	1.305
T1	0.421 ± 0.023	0.425 ± 0.026	−0.512	0.611			
T2	0.435 ± 0.026^a^	0.440 ± 0.026^a^	−0.623	0.537			
T3	0.474 ± 0.033^abc^	0.472 ± 0.02^abc^	0.252	0.802			
T4	0.430 ± 0.026	0.431 ± 0.025	−0.178	0.859			
F	14.954	13.428					
*P*	0.000**	0.000**			0.776	0.000**	0.27

*p < 0.05 **p < 0.01,a: Compared with T0, *P* < 0.05;b: Compared with T1, *P* < 0.05; c: Compared with T2, *P* < 0.05;

The respiratory parameters and circulatory parameters between the two groups at different times are presented in S1 Table. Compared with T1, the Ppeak value significantly increased in both groups at T2 and T3 (**P* *< 0.01). The PETCO_2_ values rose significantly at T3 versus T1 and T2 (*P* < 0.01).

There were no significant differences in the Ppeak, PETCO_2_, HR, and MAP between the two groups at different time points (*P* > 0.05). For HR and MAP, the main effect of time showed no statistical significance (*P* > 0.05), indicating that there were no significant changes in HR and MAP over time when the Group factor was not considered.

The Neurological and pulmonary complications are presented in S2 Table. There was no significant difference in postoperative nausea and vomiting (PONV), dizziness, headache, and postoperative pulmonary complications between the two groups (*P* > 0.05).

## Discussion

The aim of this study was to explore the effect of individualized PEEP on atelectasis, pulmonary complications, and ICP in elderly patients undergoing laparoscopic radical resection of rectal cancer by using the LUS combined with transocular measurement of the ONSD. The results showed that ΔP-guided individualized PEEP could improve intraoperative oxygenation and reduce the LUS in the early postoperative period (30 minutes after extubation), which was consistent with our initial prediction. However, there was no significant difference in the incidence of PPCs between the two groups, which may be due to the gradual recovery of atelectasis after spontaneous breathing recovery. Simultaneously, we observed that the ONSD of the two groups was wider than the basic value under the condition of Trendelenburg position and pneumoperitoneum, which was similar to the results of previous studies [17]. However, ONSD did not reach the critical value (5.5 mm) to predict intracranial pressure (ICP > 20 mmHg) [7]. There was no statistically significant difference in ONSD at different time points between the two groups, and there was also no statistically significant difference in postoperative neurological complications. This trial only observed ONSD for 1 hour in the Trendelenburg position with pneumoperitoneum, and did not observe the second and third hours. Therefore, the specific trend of ONSD increase over time was unclear.

In recent years, laparoscopic surgery has become widely accepted owing to its advantages of reduced trauma and faster recovery. However, in elderly patients undergoing general anesthesia with pneumoperitoneum and Trendelenburg positioning for a long duration, atelectasis and PPCs have increased significantly [1–3]. Previous studies have shown that appropriate PEEP can increase functional residual capacity, reduce intrapulmonary shunts, improve oxygenation and respiratory mechanics, and reduce the incidence of atelectasis and PPCs [10–11]. However, the optimal PEEP in patients receiving protective ventilation varies widely. Individualized PEEP settings guided by driving pressure can reduce the incidence of atelectasis and PPCs [12,18]. Therefore, the current study compared driving pressure-guided PEEP with fixed PEEP, and the results showed that driving pressure-guided individualized PEEP was more beneficial for reducing atelectasis.

Although CT-scan is the “gold standard” for evaluating the effect of pulmonary ventilation, it has the disadvantage of exposing patients to radiation and cannot be performed at the bedside [19]. Lung ultrasound has gradually become a potential tool for evaluating lung ventilation and lung morphology owing to its advantages of being noninvasive, not requiring radiation, and bedside operation [20–21]. The LUS is consistent with the CT classification [19,22]. The LUS can be used to quantitatively evaluate changes in pulmonary ventilation during the perioperative period [16,23]. This study showed that setting individualized PEEP according to driving pressure can reduce the LUS 30 minutes after surgery, improves oxygenation before extubation, and has a lung-protection effect, which is consistent with the results of previous studies [24].

Although individualized PEEP exhibits lung protection, whether it exerts both lung and brain protection is still controversial. Nemer found that in patients with severe traumatic brain injury and acute respiratory distress syndrome, an appropriate increase in PEEP was beneficial to oxygen supply to brain tissue [25]. In patients without craniocerebral injury, the use of low-level PEEP (5 cmH_2_O) in the Trendelenburg position did not increase intracranial pressure and could improve oxygenation [26]. However, some studies have shown that the ICP increases with increasing PEEP in patients with acute brain injury [27,28]. Therefore, a conservative PEEP of 5 cmH_2_O was used for comparison with individualized PEEP in this study. Pneumoperitoneum, Trendelenburg positioning, and PEEP are all high-risk factors for increasing ICP. Therefore, intraoperative monitoring of ICP is necessary for such high-risk patients. However, there is no unified standard for evaluating ICP in patients undergoing non-craniocerebral surgery during the operation. Studies have shown that ultrasound monitoring of ONSD is a reliable bedside method for assessing ICP [13,29], and different studies have reported different predictive values for intracranial hypertension [30–31]. The average ONSD in the Trendelenburg position and 60 minutes after pneumoperitoneum were significantly increased (4.5–5.0 mm) but did not reach the critical value of predicting intracranial hypertension (5.5 mm) [30]. Some studies have shown that when the ONSD reached 6 mm, the ICP was greater than 20 mmHg [31]. The ONSD will increase under pneumoperitoneum in the Trendelenburg position, which is similar to previous studies [17,32]. However, the increasing trend may gradually decrease because cerebral blood flow and cerebrospinal fluid may be partially compensated over time. Further studies are needed to confirm this hypothesis.

For patients without severe lung and brain diseases, compared with PEEP at 5 cmH_2_O, individualized PEEP guided by driving pressure can reduce the LUS score 30 minutes after surgery, improving oxygenation, and did not increase neurological complications. Thus, it can be safely used in laparoscopic colorectal cancer surgery and is worthy of clinical application. Since this result is derived from patients with relatively low risk of high ICP and PPCs, further verification studies are required in patients at high risk, such as those with craniocerebral injury or severe respiratory disease.

### Limitations

The limitations of this study are as follows: This was a single-center, small-sample trial. Because ICP monitoring is invasive, the indirect measurement of ICP by ONSD in this study may have individual differences and lags. The LUS is affected by the scapula on the posterior side and heart on the left side, which may make the score subjective.

## Conclusions

For patients without lung and brain diseases, individualized PEEP guided by driving pressure reduced the LUS score 30 minutes after surgery and improved oxygenation without increasing neurological complications compared with PEEP at 5 cmH₂O. Thus, it can be safely used in laparoscopic colorectal cancer surgery and is worthy of clinical application. The current findings are only based on the random samples derived from the Chinese population,and further studies in high-risk patients and other geographical areas are needed.

## Supporting information

S1 Table Comparison of respiratory and circulatory parameters between the two groups. Legend: *p < 0.05 **p < 0.01,a: Compared with T1, *P* < 0.05, b:Compared with T2, *P* < 0.05.
(TIF)

S2 Table Comparison of neurological and pulmonary complications between the two groups. Legend: *Chi-square test was used for all indicators. Abbreviations:PPCs Postoperative pulmonary complications,PONV Postoperative nausea and vomiting.
(TIF)

S1 File (DOCX)

S2 File (DOCX)

S1 Data (XLSX)
