# Supplementary figures and images for: Effect of individualized PEEP on lung ultrasound score and optic nerve sheath diameter in elderly patients undergoing laparoscopic rectal cancer surgery: A randomized controlled trial
Source: PLoS One. 2025 Aug 8;20(8):e0328067. doi: 10.1371/journal.pone.0328067 (PMC12334002; doi:10.1371/journal.pone.0328067)

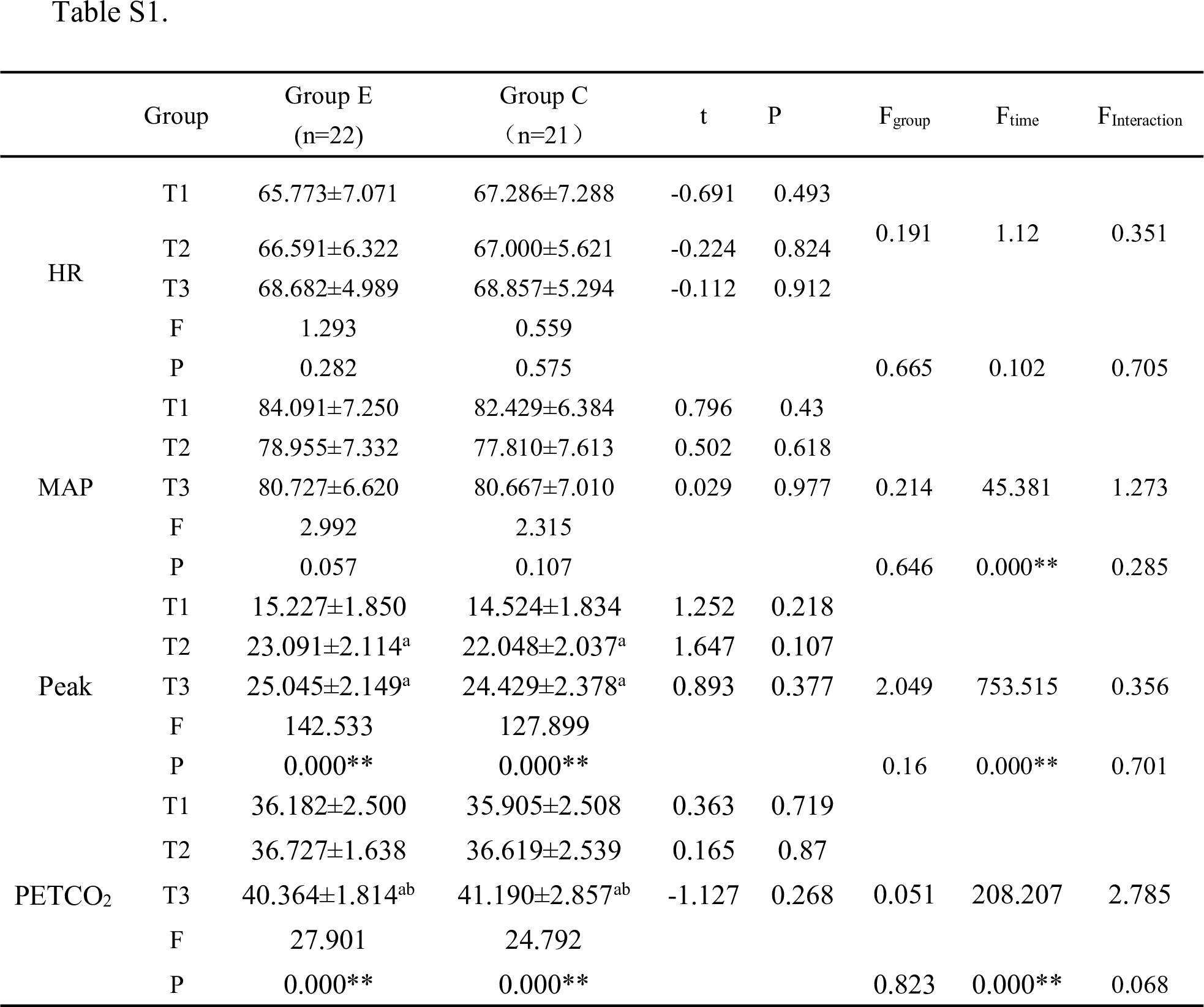

Supplement: S1 Table — Legend: *p < 0.05 **p < 0.01,a: Compared with T1, P < 0.05, b:Compared with T2, P < 0.05. (TIF) [file pone.0328067.s001.tif]

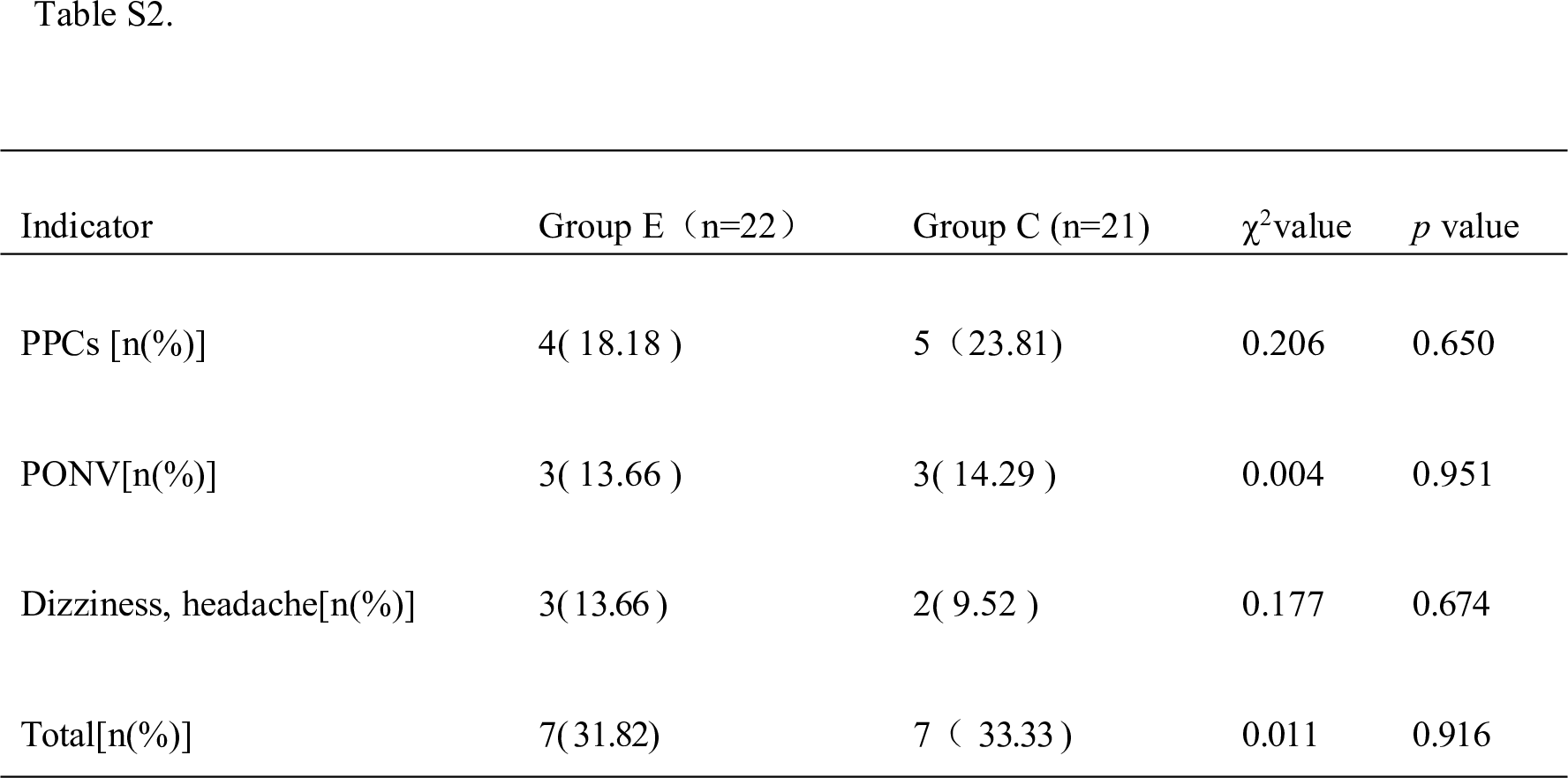

Supplement: S2 Table — Legend: *Chi-square test was used for all indicators. Abbreviations:PPCs Postoperative pulmonary complications,PONV Postoperative nausea and vomiting. (TIF) [file pone.0328067.s002.tif]
