# Supplementary material for: Effect of individualized PEEP on lung ultrasound score and optic nerve sheath diameter in elderly patients undergoing laparoscopic rectal cancer surgery: A randomized controlled trial
Source: PLoS One. 2025 Aug 8;20(8):e0328067. doi: 10.1371/journal.pone.0328067 (PMC12334002; doi:10.1371/journal.pone.0328067)
Supplement: S1 File — (DOCX) [file pone.0328067.s003.docx]

**
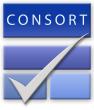
CONSORT2025 checklist of information to include when reporting a randomised trial***

| **Section/Topic** | **Item No** | **Checklist item** | **Reported on page No** |
| --- | --- | --- | --- |
| **Title and abstract** | | | |
|  | 1a | Identification as a randomised trial in the title | 1 |
|  | 1b | Structured summary of trial design, methods, results, and conclusions | 2 |
| **Open science**  Trial registration 2 Name of trial registry, identifying number (with URL) and date of registration 2  Protocol and 3 Where the trial protocol and statistical analysis plan can be accessed 4-5  statistical analysis  Plan  Data sharing 4 Where and how the individual de-identified participant data (including data dictionary), statistical code and any 10  other materials can be accessed  Funding and conflicts 5a Sources of funding and other support (e.g., supply of drugs), and role of funders in the design, conduct, analysis 10  and reporting of the trial  of interes 5b Financial and other conflicts of interest of the manuscript authors 10  **Introduction** | | | |
| Background and  Rationale  objectives | 6 | Scientific background and explanation of rationale | 3 |
|  | 7 | Specific objectives related to benefits and harms | 4-5 |
| **Methods** | | | |
| Patient and public  Involvement  Trial design | 8 | Details of patient or public involvement in the design, conduct and reporting of the tria | 3 |
|  | 9 | Description of trial design including type of trial (e.g., parallel group, crossover), allocation ratio,  and framework (e.g., superiority, equivalence, non-inferiority, exploratory) | 4 |
| Changes to trial  Protoco  Trial setting  Eligibility criteria  Intervention and  comparator | 10  11  12a  12b  13 | Important changes to the trial after it commenced including any outcomes or analyses that were not  prespecified, with reason  Settings (e.g., community, hospital) and locations (e.g., countries, sites) where the trial was conducted  Eligibility criteria for participants  If applicable, eligibility criteria for sites and for individuals delivering the interventions (e.g., surgeons,  physiotherapists)  Intervention and comparator with sufficient details to allow replication. If relevant, where additional materials  describing the intervention and comparator (e.g., intervention manual) can be accessed | 5  3  3  4-5  4-5 |
| Outcomes  Harms | 14 | Pre-specified primary and secondary outcomes, including the specific measurement variable (e.g., systolic blood pressure), analysis metric (e.g., change from baseline, final value, time to event), method of aggregation (e.g., median, proportion), and time point for each outcome | 5 |
|  | 15 | How harms were defined and assessed (e.g., systematically, non-systematically) | 5 |
| Sample size | 16a | How sample size was determined,including all assumptions supporting the sample size calculation | 6 |
|  | 16b | Explanation of any interim analyses and stopping guidelines | 5 |
| Randomisation: |  |  |  |
| Sequence  generation | 17a | Who generated the random allocation sequence and the method used | 4 |
|  | 17b | TType of randomisation and details of any restriction (e.g., stratification, blocking and block size) | 4 |
| Allocation concealment mechanism | 18 | Mechanism used to implement the random allocation sequence (e.g., central computer/telephone; sequentially  numbered, opaque, sealed containers), describing any steps to conceal the sequence until interventions were  assigned | 4 |
| Implementation | 19 | Whether the personnel who enrolled and those who assigned participants to the interventions had access to  the random allocation sequence | 4 |
| Blinding | 20a | IWho was blinded after assignment to interventions (e.g., participants, care providers, outcome assessors, data  analysts | 4 |
|  | 20b | If blinded, how blinding was achieved and description of the similarity of interventions | 4 |
| Statistical methods | 21a | Statistical methods used to compare groups for primary and secondary outcomes, including harms | 5-6 |
|  | 21b  21c  21d | Definition of who is included in each analysis (e.g., all randomised participants), and in which group  How missing data were handled in the analysis  Methods for any additional analyses (e.g., subgroup and sensitivity analyses), distinguishing prespecified from posthoc | 4-5  None  7 |
| **Results** | | | |
| Participant flow,  including flow  diagram | 22a | For each group, the numbers of participants who were randomly assigned, received intended intervention, and  were analysed for the primary outcome | 6-7 |
|  | 22b | For each group, losses and exclusions after randomisation, together with reasons | 6 |
| Recruitment  Intervention and  comparator delivery | 23a | Dates defining the periods of recruitment and follow-up for outcomes of benefits and harms | 6-7 |
|  | 23b    24a  24b | If relevant, why the trial ended or was stopped  Intervention and comparator as they were actually administered (e.g., where appropriate, who delivered the  intervention/comparator, how participants adhered, whether they were delivered as intended [fidelity])  Concomitant care received during the trial for each group | 6  4  4-5 |
| Baseline data | 25 | A table showing baseline demographic and clinical characteristics for each group | Table1 |
| Numbers analysed,  outcomes and  estimation | 26 | For each primary and secondary outcome, by group: the number of participants included in the analysis ; the number of participants with available data at the outcome time point;result for each group, and the estimated effect size and its precision (such as 95% confidence interval) ;for binary outcomes, presentation of both  absolute and relative effect size | Fig1，table1-3 |
| Outcomes and estimation |  |  |  |
| Harms  Ancillary analyses | 27  28 | All harms or unintended events in each group  Any other analyses performed, including subgroup and sensitivity analyses, distinguishing pre-specified from posthoc | 6  6-7 |
| **Discussion** | | | |
| Interpretation | 29 | Interpretation consistent with results, balancing benefits and harms, and considering other relevant evidence | 7-9 |
| Limitations | 30 | Trial limitations, addressing sources of potential bias, imprecision, generalisability, and, if relevant, multiplicity  of analyses | 10 |
