# Supplementary material for: Effect of individualized PEEP on lung ultrasound score and optic nerve sheath diameter in elderly patients undergoing laparoscopic rectal cancer surgery: A randomized controlled trial
Source: PLoS One. 2025 Aug 8;20(8):e0328067. doi: 10.1371/journal.pone.0328067 (PMC12334002; doi:10.1371/journal.pone.0328067)
Supplement: S2 File — (DOCX) [file pone.0328067.s004.docx]

**research protocol**

**Research protocol: part 1**

**Project summary**

**Objective.**Intraoperative positive end-expiratory pressure (PEEP) has been promoted for many surgical procedures. However, whether PEEP has a dual protective effect on lung and brain is controversial,and the optimal PEEP associated with dual protection is unclear. The objective was to investigate the effects of individualized PEEP on lung ultrasound score (LUS) and optic nerve sheath diameter (ONSD) in elderly patients undergoing laparoscopic surgery.

**Methods.** We enrolled 46 patients (aged 60~79 years, BMI 18~24 kg/m2, ASAI–III) ,undergoing laparoscopic radical resection of rectal cancer under general anesthesia,randomly divided into control and experimental groups. In the control group, PEEP was set at 5 cmH2O;whereas in the experimental group, 5 cmH2O PEEP was used immediately after tracheal intubation,after pneumoperitoneum was established, PEEP was gradually increased by 1 cmH2O every 10 respiratory cycles in the Trendelenburg position until ΔP reaches a minimum value or ΔP does not increase but the Ppeak has increased to 28 cmH2O. PEEP was stopped immediately when heart rate or mean arterial pressure fluctuated more than 20% of the baseline value or an arrhythmia occurred. Trendelenburg position was controlled at 30 degrees and pneumoperitoneum pressure was controlled within 12 mmHg in all patients during the operation. All patients received the same anesthesia.Anesthesia induction: cis-atracurium 0.15 mg/kg, sufentanil 0.3μg/kg, etomidate 0.2 mg/kg intravenous injection. After 5 min, the tracheal tube was inserted at the appropriate depth and fixed. Anesthesia was maintained by inhalation of 1~2% sevoflurane for sedation, intravenous pumping of 0.1~0.2 μg/kg·min remifentanil for analgesia, intermittent intravenous injection of cisatracurium (3~5 mg) as needed for muscle relaxation, and the BIS value was maintained at 40~60. The mechanical ventilation parameters setting:tidal volume was 6 mL/kg, respiratory rate was 12-18 bpm, inspiratory/expiratory ratio was 1:2. PETCO2 maintained at 35~50 mmHg. Lung recruitment maneuvers were performed every 30 min. The primary outcome was the LUS at 30 min after surgery. Secondary outcomes were ONSD at 5 min before anesthesia induction (T0), 5 min after tracheal tube insertion (T1), 5 and 60 min after Trendelenburg positioning (T2, T3), and 30 min after surgery (T4); OI and PaCO2 at T0 and before extubation at the end of the operation; and peak, PETCO2, HR, and MAP at T1-T3.In total, 43 patients completed the study.

**expected outcomes.** We hypothesized that individualized PEEP would reduce lung ultrasound scores(LUS) and the incidence of postoperative pulmonary complications(PPCs);at the same time, intracranial pressure(ICP) was not increased or even decreased.

**General information**

- Furong Bai (MS), Hong Yin(MD), Shuang Zhang(MS), Daneng Wei, Jiansheng Wang(MS), Mingliang Yi(MD)*
- Department of Anesthesiology, Chengdu Fifth People's Hospital(Affiliated Fifth People’s Hospital of Chengdu University of Traditional Chinese Medicine) , Chengdu , 611130, China
- *Mingliang Yi, MD( Corresponding author )

**Rationale & background information**

Laparoscopic radical resection of rectal cancer is more common in the elderly. However, advanced age, pneumoperitoneum, Trendelenburg positioning, and major surgery under general anesthesia are all high-risk factors for postoperative pulmonary complications (PPCs) and atelectasis [1,2,3]. Because atelectasis and PPCs can seriously affect the prognosis of patients and prolong their hospitalization duration, the prevention and reduction of PPCs and atelectasis is urgently required [4,5]. In addition, pneumoperitoneum and Trendelenburg positioning not only increase PPCs but also lead to an increase in intraocular pressure (IOP) and intracranial pressure (ICP), and can even cause neurological complications [6,7]. Studies have shown that lung protective ventilation strategy (LPVS) can reduce the incidence of PPCs [8,9]. PEEP is an essential part of the LPVS; however, there are individual differences [10]. Although individualized PEEP guided by driving pressure (ΔP) can optimize respiratory mechanics and reduce the incidence of PPCs [11,12], it is unclear whether ΔP-guided PEEP increases ICP and neurological complications. Ultrasonic detection of optic nerve sheath diameter (ONSD) is a reliable method for evaluating ICP [13], and lung ultrasound score (LUS) can evaluate lung conditions [14,15]. Although CT is the "gold standard" for evaluating the effect of pulmonary ventilation, it has the disadvantages of radiation and cannot be implemented at the bedside [19]. Lung ultrasound has gradually become a potential tool for evaluating lung ventilation and lung morphology owing to its advantages of being noninvasive, not requiring radiation, and bedside operation [20,21]. The regional LUS is consistent with the CT classification [19]. The LUS can be used to quantitatively evaluate changes in pulmonary ventilation during the perioperative period [16,22]. This study aimed to determine whether individualized PEEP guided by ΔP can achieve the dual role of lung and brain protection by monitoring ONSD and LUS scores. This study provides a scientific basis for lung and brain protective ventilation strategies in patients undergoing laparoscopic surgery.

**Study goals** **and objectives**

PEEP is one of the important measures of lung protective ventilation strategy(LPVS), but the optimal PEEP is still unclear. It is also unclear whether the relatively optimal PEEP with lung protection will affect ICP,even cause neurological complications.

1. The ideal values of PEEP is unclear,In our study, we used the individualized PEEP and fixed PEEP to compare the advantages and disadvantages.
2. we want to observe that whether individualized PEEP can reduce the LUS and the incidence of PPCs in patients undergoing surgery.
3. we want to observe that whether individualized PEEP whether has a dual protective effect on lung and brain.

**Study design**

**Inclusion criteria** were undergoing laparoscopic radical resection of rectal cancer under general anesthesia ,aged 60~79 years, BMI 18~24 kg/m2, ASAI–III) . **Exclusion Criteria** :Patients with eye and brain diseases (including high ICP and high IOP); History of eye, craniocerebral, and thoracic surgery;Preoperative respiratory diseases such as pulmonary bullae, pneumothorax, and history of pneumonia;Unable to communicate and important organ dysfunction.**Withdraw Criteria** :Surgery has changed,the experimental operation was not carried out for various reasons,the ultrasound image was disturbed.Conditions for the **termination** of the trial:

Serious adverse reactions occur during the trial,the health department requires cancel the trial. Patients were randomized into **two groups** based on random numbers: Groups E ( experimental group ,individualized PEEP guided by driving pressure,n=23) and Group C (control group ,PEEP = 5 cm H2O,n=23) .

**Methodology**

This study was approved by the Ethics Committee of Chengdu Fifth People's Hospital, document number: AF/54/2020-02.3, approval number: 2022-026 (Study) -01; It has been registered on Chinese Clinical Trial Registry(ChiCTR2200060434,1/6/2022). Informed consent was obtained from all patients who participated in the trial.From June 22022 to December 2022,46 patients undergoing laparoscopic radical resection of rectal cancer under general anesthesia, aged 60~79 years, BMI 18~24 kg/m2, ASA I~III, were randomly divided into control group and experimental group.Groups E ( experimental group ,individualized PEEP guided by driving pressure,n=23) and Group C (control group ,PEEP = 5 cm H2O,n=23) .The random numbers were generated by a computer and randomly divided into two groups in a ratio of 1:1 into opaque, sealed envelopes, which were then passed by a non-participant investigator to the anesthesiologist, who administered the anesthetic for patients. People including investigators, patients, staﬀ in the ward and postoperative care units (PACU) ) were unaware of the grouping.Postoperatively,all data were obtained by investigators who did not know the groups.

**Anesthesia protocol**. Heart rate (HR), oxygen saturation (SpO2), non-invasive blood pressure (NIBP) and bispect ral index (BIS ) value were routinely monitored in all patients after entering the operating room.Under local anesthesia, the radial artery was puncturing for pressure measurement and blood gas analysis.Anesthesia induction : cis-atracurium 0.15 mg / kg, sufentanil 0.3 ug / kg, etomidate 0.2 mg / kg intravenous injection, after 5 minutes, the tracheal tube was inserted at the appropriate depth and fixed.Anesthesia was maintained by inhalation of 1~2% sevoflurane for sedation, intravenous pumping of 0.1~0.2 ug·kg-1·min-1 remifentanil for analgesia, intermittent intravenous injection of cisatracurium 3~5 mg as needed for muscle relaxation, and BIS value was maintained at 40-60.The patient was transported back to the recovery ward after complete awakening, and all vital signs were normal.

**Mechanical ventilation protocol:** volume control mode, the tidal volume was 6ml/kg (predicted body weight ) , the respiratory rate was 12-18 times / min, inspiratory/expiratory ratio was 1:2, PETCO2 was maintain at 35~50mmHg. Lung recruitment maneuvers (RMs) was performed every 30 min.In the control group, PEEP was set at 5cmH2O; in the experimental group, 5cmH2O PEEP was used after tracheal intubation, and after pneumoperitoneum was established, PEEP was gradually increased by 1cmH2O every 10 respiratory cycles in Trendelenburg position. Until ΔP reaches a minimum value or ΔP does not increase with the increase of PEEP or ΔP does not increase but the PEAK has increased to 28cmH2O.PEEP was stopped adding immediately when heart rate or mean arterial pressure fluctuated more than 20% of the baseline value or arrhythmia occurred.

**Safety considerations**

Trendelenburg position was controlled at 30 degrees and pneumoperitoneum pressure was controlled within 12 mmHg in all patients during the operation. When systolic blood pressure or mean arterial pressure was lower than 20% of its basic value, ephedrine (3 ~ 6mg) was injected intravenously. When the HR was lower than 50 bpm, atropine (0.3 ~ 0.5 mg) was given intravenously.

**Follow-up**

The research staff collected all data until postoperative day 7 or hospital discharge (whichever came first),Patients discharged to home before day 7 without complications were considered free of complications at day 7.

**Data management and statistical analysis**

SPSS 26.0 software was used to analyse outcome data. Continuous variables were expressed as median (interquartile range) or mean ± SD. Categorical variables presented as counts (%). Data with normal distribution was evaluated using the independent sample t-test, and non-normal was calculated using the nonparametric test. Categorical variables or proportions were analyzed by chi-square test .

**Quality assurance**

Intraoperative interveners did not participate in postoperative data collection.

vestigators who were responsible for assessing all outcomes were blinded to study group assignment.

**Expected outcomes of the study**

1.we expected that individualized PEEP can reduce the LUS and the incidence of PPCs in patients undergoing surgery.

2.Individualized PEEP has a dual protective effect on lung and brain.

**Dissemination of results and publication policy**

Furong Bai will take the lead in publication and Yi Hong Yin,Jiansheng Wang, Shuang Zhang, and Ming-liang will be acknowledged in publications.

**Duration of the project**

Data collection began in June 2022 and ended in December 2022;

Data analysis began in February 2022 and ended in March 2022;

The first draft of the thesis is from April 2022 to May 2022;

The final paper completed in July 2022;

**Problems anticipated**

Since some patients may have subcutaneous emphysema during the operation, the lung ultrasound evaluation would be affected, so,we may have to expand the sample size.

**Project management**

Conceptualization: Furong Bai, Hong Yin, Shuang Zhang, Daneng Wei, Jiansheng Wang, Mingliang Yi.Data curation:Furong Bai, Hong Yin, Jiansheng Wang, Mingliang Yi .Formal analysis: Shuang Zhang, Daneng Wei.Investigation: Furong Bai,Mingliang Yi.Methodology: Furong Bai, Hong Yin, Shuang Zhang, Daneng Wei,Mingliang Yi.Project administration: Furong Bai,Mingliang Yi.Resources: Furong Bai,Hong Yin,Mingliang Yi.Writing -original draft: Furong Bai.

**Ethics**

Institutional review board approval was obtained from Ethics committee of Chengdu Fifth People's Hospital (Ethical Number: 2022-026 (Study) -01).And obtain a standard version of informed consent forms from Ethics committee.

**Informed consent forms**

The approved version of the protocol must have copies of informed consent forms (ICF),we used the local language (Chinese) to administe.

**References (of literature cited in preceding sections)**

1. Jo YY, Lee KC, Chang YJ, et al. Effects of an Alveolar Recruitment Maneuver During Lung Protective Ventilation on Postoperative Pulmonary Complications in Elderly Patients Undergoing Laparoscopy[J]. Clin Interv Aging. 2020;15:1461-1469.doi:10.2147/CIA.S264987.
2. Kaufmann K,Heinrich S.Minimizing postoperative pulmonary complications in thoracic surgery patients[J]． Curr Opin Anaesthesiol，2021,34( 1) :13-19．doi: 10.1097/ACO.0000000000000945.
3. Fernandez-Bustamante Ana, Frendl Gyorgy, Sprung Juraj, et al.Postoperative Pulmonary Complications, Early Mortality, and Hospital Stay Following Noncardiothoracic Surgery: A Multicenter Study by the Perioperative Research Network Investigators[J]. JAMA Surgery. 2017, 152(2):157-166.doi: 10.1001/jamasurg.2016.4065.
4. Qi-Wen Deng,Wen-Cheng Tan,Bing-Cheng Zhao, et al. Intraoperative ventilation strategies to prevent postoperative pulmonary complications: a network meta-analysis of randomised controlled trials[J]. British Journal of Anaesthesia,2020; 124:324-335.doi: 10.1016/j.bja.2019.10.024.
5. Thomas Bluth,Ary Serpa Neto,Marcus J. et al. Effect of Intraoperative High Positive End-Expiratory Pressure (PEEP) With Recruitment Maneuvers vs Low PEEP on Postoperative Pulmonary Complications in Obese Patients: A Randomized Clinical Trial[J].2019; 321:2292-2305.doi: 10.1001/jama.2019.7505.
6. Goel Nitesh,Chowdhury Itee,Dubey Jitendra, et al. Quantitative rise in intraocular pressure in patients undergoing robotic surgery in steep Trendelenburg position: A prospective observational study[J]. Journal of Anaesthesiology Clinical Pharmacology,2020,36(4):546-551.doi: 10.4103/joacp.JOACP_96_20.
7. CHEN K，WANG L，WANG Q，et al. Effects of pneumoperito-neum and steep Trendelenburg position on cerebral hemodynamics during robotic assisted laparoscopic radical prostatectomy：A randomized controlled study［J］. Medicine（Baltimore）,2019,98(21):e15794.doi: 10.1097/MD.0000000000015794.
8. Futier Emmanuel,Constantin Jean-Michel,Paugam-Burtz Catherine. et al.De Jong Audrey,Bazin Jean-Etienne,Pereira Bruno,Jaber Samir. A trial of intraoperative low-tidal-volume ventilation in abdominal surgery.[J]. The New England journal of medicine,2013; 369:428-437.doi: 10.1056/NEJMoa1301082.
9. Young Christopher C,Harris Erica M,Vacchiano Charles,et al. Lung-protective ventilation for the surgical patient: international expert panel-based consensus recommendations.[J]. British journal of anaesthesia,2019,123(6):898-913.doi: 10.1016/j.bja.2019.08.017.
10. Pereira Sérgio M,Tucci Mauro R,Morais Caio C A, et al. Individual Positive End-expiratory Pressure Settings Optimize Intraoperative Mechanical Ventilation and Reduce Postoperative Atelectasis.[J]. Anesthesiology,2018,129(6):1070-1081.doi: 10.1097/ALN.0000000000002435.
11. Fernandez-Bustamante A, Sprung J, Parker RA, et al.Individualized PEEP to optimise respiratory mechanics during abdominal surgery: a pilot randomised controlled trial[J]. Br J Anaesth. 2020 Sep;125(3):383-392.doi: 10.1016/j.bja.2020.06.030.
12. Park MiHye,Ahn Hyun Joo,Kim Jie Ae, et al. Driving Pressure during Thoracic Surgery: A Randomized Clinical Trial[J]. Anesthesiology,2019;130(3):385-393.doi: 10.1097/ALN.0000000000002600.
13. SHIN S S，HUISMAN T A G M，HWANG M． Ultrasound imaging for traumatic brain injury[J].J Ultrasound Med,2018,37( 8) : 1857-1867．doi: 10.1002/jum.14547.
14. Finance Julie,Zieleskewicz Laurent,Habert Paul, et al.Low Dose Chest CT and Lung Ultrasound for the Diagnosis and Management of COVID-19[J]. Journal of Clinical Medicine,2021,10(10):2196. doi: 10.3390/jcm10102196.
15. Mongodi Silvia,De Luca Daniele,Colombo Andrea, et al. Quantitative Lung Ultrasound: Technical Aspects and Clinical Applications[J]. Anesthesiology,2021,134(6):949-965.doi: 10.1097/ALN.0000000000003757.
16. Généreux V，Chassé M，Girard F，et al． Effects of positive endexpiratory pressure /recruitment manoeuvres compared with zero end-expiratory pressure on atelectasis during open gynaecological surgery as assessed by ultrasonography: a randomised controlled trial[J].Br J Anaesth,2020,124( 1) :101-109．doi: 10.1016/j.bja.2019.09.040.
17. Vitiello L, De Bernardo M, Rosa N. Regarding "Effect of Pneumoperitoneum and Patient Positioning on Intracranial Pressures during Laparoscopy: A Prospective Comparative Study"[J]. J Minim Invasive Gynecol. 2019;26(6):1199. doi:10.1016/j.jmig.2019.05.025
18. Mini G, Ray BR, Anand RK, et al. Efect of driving pressure-guided positive end-expiratory pressure (PEEP) titration on postoperative lung atelectasis in adult patients undergoing elective major abdominal surgery: a rand‑omized controlled trial[J]. Surgery. 2021;170(1):277-283. doi: 10.1016/j.surg.2021.01.047.
19. Chiumello D，Mongodi S，Algieri I，et al． Assessment of lung aeration and recruitment by CT scan and ultrasound in acute respiratory distress syndrome patients[J]. Crit Care Med,2018,46( 11):1761-1768.doi: 10.1097/CCM.0000000000003340.
20. Senniappan K，Sreedhar R，Babu M，et al． Bedside lung ultrasound for postoperative lung conditions in cardiothoracic intensive care unit: diagnostic value and comparison with bedside chest roentgenogram[J].Anesth Essays Res，2019,13( 4) :649-653．doi: 10.4103/aer.AER_125_19.
21. Dransart-Rayé O，Roldi E，Zieleskiewicz L，et al． Lung ultrasound for early diagnosis of postoperative need for ventilatory support: a prospective observational study[J]．Anaesthesia,2020,75( 2) :202-209．doi: 10.1111/anae.14859.
22. Jang YE，Ji SH，Kim EH，et al． Effect of regular alveolar recruitment on intraoperative atelectasis in paediatric patients ventilated in the prone position: a randomised controlled trial[J]. Br J Anaesth,2020,124( 5) :648-655．doi: 10.1016/j.bja.2020.01.022.
23. Xu Q, Guo X, Liu J, et al. Effects of dynamic individualized PEEP guided by driving pressure in laparoscopic surgery on postoperative atelectasis in elderly patients: a prospective randomized controlled trial[J]. BMC Anesthesiol. 2022;22(1):72. doi: 10.1186/s12871-022-01613-9.
24. Nemer SN, Caldeira JB, Santos RG, et al. Effects of positive end-expiratory pressure on brain tissue oxygen pressure of severe traumatic brain injury patients with acute respiratory distress syndrome: A pilot study. [J].J Crit Care. 2015 Dec;30(6):1263-6. doi: 10.1016/j.jcrc.2015.07.019.
25. You Ann Hee,Song Young,Kim Do-Hyeong,et al. Effects of positive end-expiratory pressure on intraocular pressure and optic nerve sheath diameter in robot-assisted laparoscopic radical prostatectomy: A randomized, clinical trial.[J]. Medicine,2019,98(14):e15051.doi: 10.1097/MD.0000000000015051.
26. Robba C, Ball L, Nogas S, et al. Effects of Positive End-Expiratory Pressure on Lung Recruitment, Respiratory Mechanics, and Intracranial Pressure in Mechanically Ventilated Brain-Injured Patients. [J].Front Physiol. 2021 Oct 18;12:711273.
27. Bala Renu,Kumar Rajesh,Sharma Jyoti. A study to evaluate effect of PEEP and end-tidal carbon dioxide on optic nerve sheath diameter.[J].Indian journal of anaesthesia,2019,63(7):537-543.doi: 10.4103/ija.IJA_861_18.
28. Koziarz A, Sne N, Kegel F, et al. Bedside Optic Nerve Ultrasonography for Diagnosing Increased Intracranial Pressure: A Systematic Review and Meta-analysis. [J].Ann Intern Med. 2019;171(12):896-905. doi:10.7326/M19-0812
29. Sahu Seelora,Panda Nidhi,Swain Amlan,et al. Optic Nerve Sheath Diameter: Correlation With Intra-Ventricular Intracranial Measurements in Predicting Dysfunctional Intracranial Compliance.[J]. Cureus,2021,13(1):e13008.doi: 10.7759/cureus.13008.
30. ＲOBBA C，DONNELLY J，CAＲDIM D，et al． Optic nerve sheath diameter ultrasonography at admission as a predictor of intracranial hypertension in traumatic brain injured patients: a prospective observational study［J］.J Neurosurg，2019,132( 4) :1279-1285．doi: 10.3171/2018.11.JNS182077.
31. Kim MS，Bai SJ，Lee JＲ，et al． Increase in intracranial pressure during carbon dioxide pneumoperitoneum with steep trendelenburg positioning proven by ultrasonographic measurement of optic nerve sheath diameter[J]．J Endourol,2014，28( 7) : 801-806．doi: 10.1089/end.2014.0019.

**Research protocol: part 2**

**Budget**

Ultrasound assessment does not cost extra,Only arterial blood gas analysis costs about 30 dollars each person which were been undertaken by Furong Bai.

**Curriculum Vitae of investigators**

Ming-Liang Yi:professor,Postgraduate tutor,the main research direction is organ protection.

Fu-Rong Bai:Master,the main research direction is lung protection.

Hong-Yin: professor,Postgraduate tutor, the main research direction is lung protection.

Jiansheng Wang:Master,engaged in clinical work for more than 15 years.

Shuang Zhang:Master, engaged in clinical work for more than 5 years.
